# Assessing quality of life in a randomized clinical trial: Correcting for missing data

**DOI:** 10.1186/1471-2288-9-28

**Published:** 2009-04-30

**Authors:** Nina Gunnes, Taral G Seierstad, Steinar Aamdal, Paal F Brunsvig, Anne-Birgitte Jacobsen, Stein Sundstrøm, Odd O Aalen

**Affiliations:** 1 Department of Biostatistics, University of Oslo, P.O. Box 1122 Blindern, N-0317 Oslo, Norway; 2 Department of Oncology, The Norwegian Radium Hospital, Montebello, N-0310 Oslo, Norway; 3 Department of Oncology, St. Olavs Hospital, Olav Kyrres gate 17, N-7006 Trondheim, Norway

## Abstract

**Background:**

Health-related quality of life is a topic of current interest. This paper considers a randomized phase III study of radiation therapy with concurrent chemotherapy (docetaxel) versus radiation therapy alone in non-small cell lung cancer, stage III A/B. Longitudinal data on quality of life have been obtained through repeated administration of a multi-item questionnaire (EORTC QLQ-C30) developed by the European Organisation for Research and Treatment of Cancer. Missingness in the data is owing to patients having failed to complete the questionnaire at some of the scheduled filling-in times.

**Methods:**

We have analysed a monotone (in terms of missingness) subset of the data as regards estimation of the mean score of a summary measure of self-reported quality of life in a hypothetical drop-out-free population at different points in time. Missingness is a difficult issue of great importance. We have therefore chosen to compare three different methods that are relatively easy to implement: the linear-increments method, the inverse-probability-weighting method and the Markov-process method. Single imputation has been applied in a supplementary analysis to fill in for all the non-consecutive missing score values prior to the execution of the estimation procedure.

**Results:**

For the response in focus, the observed mean score at a certain time is larger than the estimated mean scores, which implies that the true mean score is easily overestimated unless the missingness is appropriately adjusted for. Comparison of the treatment arms shows a significant difference in mean score at the end of treatment.

**Conclusion:**

Use of proper methodology developed for analysing data subject to missingness is necessary to reduce potential estimation bias. The quality of life of patients receiving radiation therapy with concurrent chemotherapy (docetaxel) appears somewhat worse than that of patients receiving radiation therapy alone in the period during which treatment is given. The conclusions are robust for the choice of statistical methods.

## Background

Quality of life (QoL) is a rather complex multi-dimensional concept that can be defined as the degree of well-being felt by an individual [1]. It is commonly divided into two different components: a *physical *component and a *psychological *component. The former includes diet, health, etc., while the latter involves different emotional states such as worry, fear, sorrow and happiness. In health care it is very important to consider QoL in the course of a treatment evaluation. Since QoL is based on subjective assessments, it is not easily quantifiable, as opposed to more concrete measures like e.g. weight and blood pressure.

Health-related QoL has been an area of research over the past 20 years, and several international validated self-report questionnaires have been developed in this regard and used in longitudinal studies.

A longitudinal study involves time-*discrete *observation of time-*continuous *processes, where measurements of the variables of interest are taken at consecutive points in time. These times are often represented by so-called study waves; wave 1 represents the time at which the first set of measurements is taken, wave 2 represents the time at which the second set of measurements is taken, and so on. A problem arises when study participants die, are lost to follow-up or for other reasons fail to contribute all of the planned sets of measurements. This resulting incompleteness of data is a challenge to the analyst, and it may lead to biased results if it is not taken into account in the statistical analysis and adjusted for in an appropriate way. The missingness is said to be of a *monotone *kind if a subject that fails to contribute measurements at a certain study wave, also fails to contribute measurements at all of the subsequent waves. Otherwise, the missingness is said to be of a *non*-*monotone *kind.

In this paper we consider a monotone (in terms of missingness) subset of longitudinal measurements of QoL. The data are obtained from a randomized phase III study of radiation therapy with concurrent chemotherapy versus radiation therapy alone in non-small cell lung cancer (NSCLC), stage III A/B. Location of the randomization centre for this international multi-centre study was at The Norwegian Radium Hospital in Oslo, Norway. The clinical trial was approved by the Hospital Review Board, the Regional Ethics Committee and the Norwegian Medicines Agency. A total of 261 patients diagnosed with NSCLC, stage III A (inoperable) or stage III B, were included in the study between April 2000 and June 2006. Twelve of the initially included patients were later excluded from the study for not fulfilling the inclusion criteria. The final study sample thus consisted of 249 patients (157 men and 92 women) from Denmark, Finland, Norway and Sweden. The study medication administration was divided into two different treatment arms: arm A (study arm) and arm B (standard arm). The former involved six weeks of radiation therapy, given five days a week, combined with weekly infusion of the cytotoxic drug docetaxel (Taxotere^®^), whereas the latter involved solely six weeks of radiation therapy. Upon inclusion, the patients were independently randomized to one of the two treatment arms; 119 (48%) of the patients were randomized to arm A, and 130 (52%) of the patients were randomized to arm B. Also, prior to inclusion of its first patient, each involved centre had to decide whether two courses of induction chemotherapy would be given before start of treatment, in which case the same regimen would be used for all patients included by that particular centre. Induction chemotherapy involves initial treatment by giving the patient standard chemotherapy before the start of radiation therapy with the intention to reduce the volume of the tumour (downstaging) in such a way that the radiation area is reduced.

The primary objective of the study was to compare the survival time of radiation therapy combined with docetaxel versus radiation therapy alone, and the secondary objective was to compare the time to progression and QoL in the two treatment groups. Validated self-report, multi-item questionnaires have been developed by the European Organisation for Research and Treatment of Cancer (EORTC) in order to assess the QoL of cancer patients participating in clinical trials. Translated versions of the EORTC QLQ-C30 [2], supplemented by a lung cancer module, were administrated to the patients at a pre-specified set of times during follow-up: immediately before start of treatment (control week 0), at the end of treatment (control week 6), six weeks after end of treatment (control week 12), and then every 12 weeks until death, drop-out or closure of the study in January 2009. The EORTC QLQ-C30 includes 30 items in the form of questions regarding a patient's symptoms, health and competency to perform various daily life tasks, and in that way it covers and reflects different generic aspects of QoL. Each item is answered by circling the number corresponding to the pre-coded response option that best applies. Nineteen of the patients (9 in arm A and 10 in arm B) started induction therapy at the time of randomization. The timing of the questionnaires for these patients differed from protocol, and hence, their answers have been discarded.

We have focused on item 30 in the EORTC QLQ-C30, which is given by the following question: "How would you rate your overall quality of life during the past week?". This can be regarded as a summary measure of QoL, taking integer score values in the range from 1 to 7, where scores of 1 and 7 correspond to 'very poor' and 'excellent', respectively. That is, the higher the score value, the higher the QoL as measured by this particular item. Our aim has been to estimate the mean score of item 30 in a hypothetical drop-out-free population in which every subject contributes all planned sets of measurements. Ignoring missingness present in the data might lead to biased mean score estimates, and so we have made use of different adjusting techniques. It is not obvious whether one should adjust for all missing observations, including those due to death, or whether one should only consider surviving patients. The former corresponds to analysing an *immortal *cohort, while the latter corresponds to analysing a *mortal *cohort [3]. On the surface, the mortal cohort analysis seems more reasonable, but in reality one may get a false impression of the relationship between treatments. For instance, it may be the case that one treatment improves survival, but at the cost of QoL. Hence, the treatment that is better in terms of survival may, precisely because of this advantage, come out worse in terms of QoL. Therefore, the immortal cohort analysis may be worth considering. The procedure of correcting for all missing observations, without regard to cause, can be quite sensible in many circumstances and give a more fair comparison of treatments. This will be our main approach since we indeed wish to compare arm A and arm B as regards QoL.

One further note should be made regarding adjusting for mortality. In survival studies there is usually an amount of censoring due to subjects entering the study late and thus being under follow-up for just a short period of time. In these cases one will not know when death takes place, and so distinguishing between death and missingness due to other causes may not be feasible. Hence, adjusting for all missing observations may be the most clear-cut approach. However, for the disease studied here, mortality is high, and most patients have been followed until death. Therefore, we have also performed a mortal cohort analysis, where patients are removed from the study at their known death times, and we have compared this with the other analysis.

The employed methodology includes three methods that rest on different assumptions. Merely using one method could then result in wrong conclusions if the relevant assumptions were not to be true. By using two or three methods, the conclusions will be more certain and robust when the respective results agree. The methodology has been implemented using the programming language Matlab^® ^[4].

## Methods

In this section we introduce the statistical framework used for analysing longitudinal data subject to monotone missingness with regard to estimation of the mean of a time-continuous, discrete-valued response variable.

### Notation

Consider a longitudinal study of a time-continuous response process , taking only discrete values, and some time-continuous covariate processes , which can take both discrete and continuous values. In accordance with Diggle et al. [5] and Gunnes et al. [6], we refer to the variable (*t*) as the *hypothetical *response at time *t*, that is, the response that would have been recorded had the subject, possibly contrary to fact, contributed a measurement at this time. In the same way, we let (*t*) be the hypothetical covariates at time *t*. Measurements of the response and covariates are scheduled for a pre-specified set of ordered times *t*_1_,..., *t*_*τ*_, where *τ *is the total number of measurement occasions. We assume that the data are subject to monotone missingness, and the predictable time-continuous response indicator process is denoted by *R*. The term 'predictable' means that the value of *R*(*t*) is known at time *t*-, i.e. right before *t*. We set *R*(*t*) equal to 1 if the subject has contributed all planned measurements of the response and covariates up to, and including, time *t*. Otherwise, we set *R*(*t*) equal to 0. Further, we write *Y*(*t*_1_),..., *Y *(*t*_*T*_) for the *observed *responses, where *T *≤ *τ *is the total number of measurement sets the subject gives rise to. Correspondingly, we write *X*(*t*_1_),..., *X *(*t*_*T*_) for the observed covariates.

The specification of the missingness and censoring schemes presented below is based on the history of the observed and unobserved processes. Following the notation of Gunnes et al. [6], the past history and strict past history of the hypothetical time-continuous response process  and covariate processes  at time *t *are written  and , respectively. In the same way, ℛ_[*t*] _denotes the past history of the time-continuous response indicator process *R *at time *t*, and ℛ_(*t*) _denotes its strict past. Note that since *R *is predictable, we have ℛ_[*t*] _= ℛ_(*t*)_. If we restrict these histories to the scheduled measurement times, we set  and make an equivalent definition of . Further, we let  and  denote the past history and strict past history, respectively, of the time-discrete observed response and covariate processes, where .

### Missingness and censoring schemes

The methodology that we have made use of in our work is based on some assumptions regarding the response indicator process *R*.

The *missingness completely at random *(MCAR) condition [[7], chapter 1.3] states that the response indicator process is independent of the hypothetical response process  and covariate processes :(1)

In other words, knowledge of all realizations of the response and covariate variables does not influence the dropout probability.

When the *missingness at random *(MAR) condition [8] is fulfilled, the response indicator process only depends on the observed data:(2)

This means that the probability of dropping out is unaffected by response and covariate values that are not observed. MAR is guaranteed by insisting that the response indicator process depends solely on previously observed responses and covariates. On the other hand, if the response indicator process depends on unobserved data, we have *missingness not at random *(MNAR).

The *continuous-time independent censoring *(CTIC) condition [9] can be defined as follows:(3)

where  is shorthand for . A sufficient, but not necessary, condition for CTIC is  for every time *t*. This allows *R*(*t*) to depend on any aspect of the past of  and  but for the current infinitesimal  and .

A stronger condition than CTIC is the *discrete-time independent censoring *(DTIC) condition, which recognizes that longitudinal data are measured in discrete time:(4)

Thus, it places constraints on the expected value of the increment  of the hypothetical response. A sufficient condition for DTIC is  for each time *t*_*k*_. This implies that *R*(*t*_*k*_) may only depend on  and  until time *t*_*k*-1_, and not on the interval (*t*_*k*-1_, *t*_*k*_) [6].

The DTIC condition may seem somewhat unrealistic, but it corresponds to what can actually be observed. Clearly, we cannot correct for the unobserved development within an interval.

### The linear-increments method

The linear-increments (LI) method postulates linear models for the increments of the hypothetical response process  at different times. This was first proposed by Diggle et al. [5] for continuous-valued response variables. Gunnes et al. [6] discuss the LI technique for discrete-valued response variables, for which the model at time *t*_*k *_is given by(5)

Here, the predictors (*t*_*k*_) are functions of the strict past history , and *β*(*t*_*k*_) are the corresponding regression functions. Following Diggle et al. [5] and Gunnes et al. [6], we make the definitions (0) := 0 and (0) := 0. The former leads to , where *t*_1 _> 0 is the time at which the first set of measurements are taken.

Assuming DTIC, linear models are induced on the increments  of the observed data as well:(6)

where . The regression functions *β*(*t*_*k*_) are the same as for the hypothetical data, and they are estimated for each time *t*_*k *_using ordinary least squares regression.

For every subject, the mean hypothetical response at time *t*_*k *_is estimated by replacing the regression functions with the ordinary least squares estimates  and then, recursively, inserting previously obtained estimates into Equation (5) and calculating the cumulative sum. Finally, an estimate of the population mean  of the hypothetical response at time *t*_*k *_is given by the arithmetic average of all individual estimated mean hypothetical responses. The detailed procedure is given by Gunnes et al. [6].

### The inverse-probability-weighting method

As the name suggests, the inverse-probability-weighting (IPW) method involves weighting the observed responses at a certain time by the inverse of the respective probabilities of measurements being taken, and thus, creating a pseudo-population where no data are missing. Following Gunnes et al. [6], we let *ρ*(*t*_*k*_) = Pr{*R*(*t*_*k*_) = 1} be the probability that the subject contributes measurements of the variables of interest at time *t*_*k*_, and we set *ρ*(*t*_1_) ≡ 1 for all subjects. Further, we let  be the conditional probability that the subject contributes a set of measurements at time *t*_*k*_, given that a set of measurements was contributed at *t*_*k*-1_. Under the assumption of monotone missingness, the probability that the subject contributes a set of measurements at time *t*_*k *_≥ *t*_2 _is given by(7)

If the MAR condition is fulfilled, the unknown conditional probabilities  can be estimated in a preliminary pooled logistic regression analysis [3,6]:(8)

Here, the predictors *Z*(*t*_*k*_) are functions of the time *t*_*k *_and , and *α *are the corresponding time-independent regression coefficients. Subject-specific weights *w*(*t*_*k*_) are found by taking the inverse of the respective estimated measurement probabilities . We have used "stabilized" weights [[10], page 562]  to reduce the variability of the estimates. Here,  is the estimated probability that a set of measurements is taken at time *t*_*k*_, calculated by including only baseline covariates in the logistic model given in Equation (8).

Finally, the population mean  of the hypothetical response at time *t*_*k *_is estimated by a weighted arithmetic average of all observed responses:(9)

where *Y*_*i*_(*t*_*k*_) denotes the observed response of subject *i *at time *t*_*k*_, with corresponding weight *w*_*i*_(*t*_*k*_), and *I*(*t*_*k*_) is the set of subjects of which measurements are taken at *t*_*k *_[6].

### The Markov-process method

The Markov-process (MP) method [6] is based on an assumption that the hypothetical response process  is a Markov process with a finite state space  = {1,..., *U*}, where *U *∈ ℕ is some natural number. Each state represents a certain value of the response. Assuming monotone missingness, we let  be the number of observed subjects in state *u *at time *t*_*k*-1 _and in state *v *at time *t*_*k*_. Here, *S*_*i*_(*t*_*k*_) denotes the state occupied, that is, the response value attained, by subject *i *at time *t*_*k*_, and *I *(*t*_*k*_) is the set of subjects of which measurements are taken at *t*_*k*_.

If the DTIC condition is fulfilled, the discrete analogue  of the time-continuous

Aalen-Johansen estimator [11] of the transition probability matrix at time *t*_*k *_≥ *t*_2 _is given by(10)

where , and  equals the *U*-dimensional identity matrix [6]. The estimated occupation probability of state *v *at time *t*_*k *_is(11)

Here,  is the empirical proportion of *n *subjects occupying state *u *at time *t*_1_. Finally, the population mean  of the hypothetical response at time *t*_*k *_is given by a weighted sum of the estimated state occupation probabilities:(12)

where *c*_*u *_denotes the value of the hypothetical response corresponding to occupation of state *u *[6].

### Single imputation

Subjects participating in longitudinal studies occasionally fail to contribute measurements of the variables of interest while under follow-up. This can result in a considerable loss of information, especially when the employed methodology is developed for analysing monotone (in terms of missingness) subsets of the data. In order to be able to utilize more of the available data, a feasible approach is to use single imputation to fill in for all non-consecutive, i.e. isolated, missing values that are directly preceded and succeeded by observed values. Thus, a new "artificial" and more complete monotone (in terms of missingness) subset of the data is created. (Multiple imputation has not been used here since the added complexity was not deemed necessary.)

In a supplementary analysis we have chosen to impute a non-consecutive missing value at time *t*_*k *_by the arithmetic average of the two corresponding adjacent observed values at times *t*_*k*-1 _and *t*_*k*+1_. That is, for instance, if a subject contributes a measurement of value 4 at a certain time, fails to contribute a measurement at the following time and then contributes a measurement of value 6 at the next time, the missing value in between the two observed ones is imputed by (4 + 6)/2 = 5.

The MP method is currently developed only for integer-valued responses or responses that can be cast in this form. Since the non-consecutive missing values in some cases may be imputed by decimal numbers, i.e. non-integers, we have not calculated the MP estimates when single imputation has been applied prior to the data analysis.

During treatment and the first couple of weeks following end of treatment, the scores reported by the patients randomized to arm A changed considerably, and so, imputation of missing values in this period using the technique described above would be inappropriate and might lead to biased mean score estimates. Therefore, missing values at the first three scheduled filling-in times of the EORTC QLQ-C30, that is, control weeks 0, 6 and 12, have not been imputed for either of the treatment arms.

## Results

As previously mentioned, item 30 in the EORTC QLQ-C30 has been the response in focus. This item deals with the overall QoL of a patient during the past week. The observation of the response process is discrete (in time), corresponding to the filling in of the questionnaire.

It is reasonable to believe that the expected increment of a discrete-valued response at time *t*_*k *_will depend on its previous value at time *t*_*k*-1_, as will the probability of contributing a response measurement at time *t*_*k*_. In addition, we assume that sex, treatment arm and whether or not induction therapy was given will affect the response process as well as the response indicator process. In consequence, the following covariates have been included in the linear regression model of the LI method: the previous score, indicator for being a woman, indicator for being randomized to arm A and indicator for having received induction chemotherapy. Further, the following covariates have been included in the pooled logistic regression model of the IPW method: indicators for the possible values of the previous score, time, indicator for being a woman, indicator for being randomized to arm A and indicator for having received induction chemotherapy. (Note that in the analysis where single imputation has been applied, the previous score, instead of indicators for the possible values of the previous score, has been included in the pooled logistic regression model of the IPW method. The reason for this is that then the previous score value may actually be a decimal number and not an integer in the range 1–7.)

Two corresponding immortal cohort analyses have been performed using the three estimation methods. Single imputation was not applied in the first analysis, whereas in the second analysis it was applied. For comparison, a mortal cohort analysis, without applying single imputation, has also been performed using the LI method.

Because of the assumption of monotone missingness, only a selection of the score values in the original data set are considered to be observed in a specific analysis, and the remaining score values are thus regarded as missing. All our analyses are restricted to 198 patients (98 in arm A and 100 in arm B) whose respective score values at control week 0, that is, immediately before start of treatment, are available. Keep in mind that in the analysis where single imputation has been applied, some of the observed score values, with respect to monotone missingness, are actually missing values that have been imputed.

### Without single imputation

**Figure 1:**
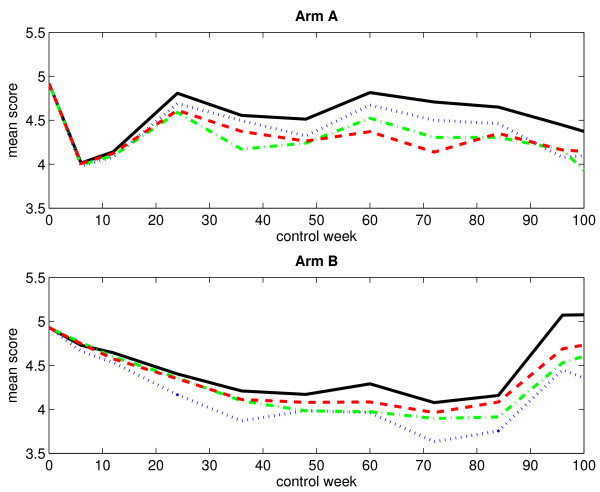
**Observed and estimated mean scores for an immortal cohort (without single imputation)**. The figure displays the observed and estimated mean scores for arm A (upper panel) and arm B (lower panel) when considering an immortal cohort. Single imputation has not been applied. The black solid-line curve corresponds to the arithmetic average of the observed score values, the blue dotted-line curve corresponds to the IPW method, the green dash-dotted-line curve corresponds to the LI method, and the red dashed-line curve corresponds to the MP method.

**Figure 2:**
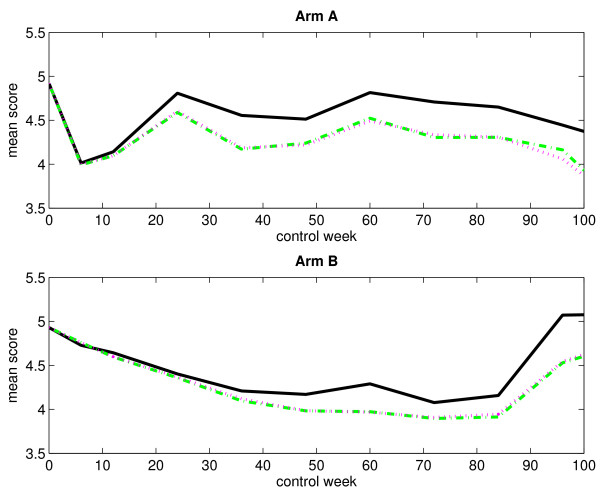
**Observed and estimated mean scores for a mortal cohort (without single imputation)**. The figure displays the observed and estimated mean scores for arm A (upper panel) and arm B (lower panel) when considering a mortal cohort. Single imputation has not been applied. The black solid-line curve corresponds to the arithmetic average of the observed score values, the magenta dotted-line curve corresponds to the LI method, and the green dash-dotted-line curve corresponds to the LI method when considering an immortal cohort (for comparison).

Table 1 presents the numbers of observed score values, with respect to monotone missingness, for both treatment arms at different control weeks. The corresponding numbers of missing score values are presented in Table 2. Obviously, the numbers of observed score values decrease over time as the patients fail to answer the current question. In the same way, the numbers of missing score values increase over time. Figure 1 displays the mean score estimates, plotted against time, for both treatment arms when considering an immortal cohort. In the plot corresponding to arm A, we notice a rapid decline in the curves right after start of treatment. At control week 6, they reach a low before increasing. This sudden dip at the end of treatment is most likely due to some of the adverse effects of chemotherapy, such as nausea and discomfort, which generally lead to low score values. The curves fluctuate somewhat after control week 24. In contrast, the curves in the plot corresponding to arm B fall gradually. They begin to rise again at control week 84. Figure 2 displays the LI estimates of the mean score, plotted against time, for both treatment arms when considering a mortal cohort. We observe no important differences between the immortal cohort analysis and the mortal cohort analysis as regards estimation of the mean score using the LI method.

**Table 1:** Numbers of observed score values (without single imputation).

	Control week										
	
	0	6	12	24	36	48	60	72	84	96	108
Arm A	98	73	64	52	45	39	27	24	20	18	13

Arm B	100	81	73	67	62	47	31	26	19	14	12

The table presents the numbers of observed score values, with respect to monotone missingness, for arm A and arm B at different control weeks. Single imputation has not been applied.

**Table 2:** Numbers of missing score values (without single imputation).

		Control week										
		
		0	6	12	24	36	48	60	72	84	96	108
Arm A	Death	0	3	9	23	30	37	48	56	61	66	72
	
	Other causes	0	22	25	23	23	22	23	18	17	14	13

Arm B	Death	0	0	3	13	20	29	42	50	59	62	69
	
	Other causes	0	19	24	20	18	24	27	24	22	24	19

The table presents the numbers of missing score values, with respect to monotone missingness, due to death and other causes for arm A and arm B at different control weeks. Single imputation has not been applied.

**Figure 3:**
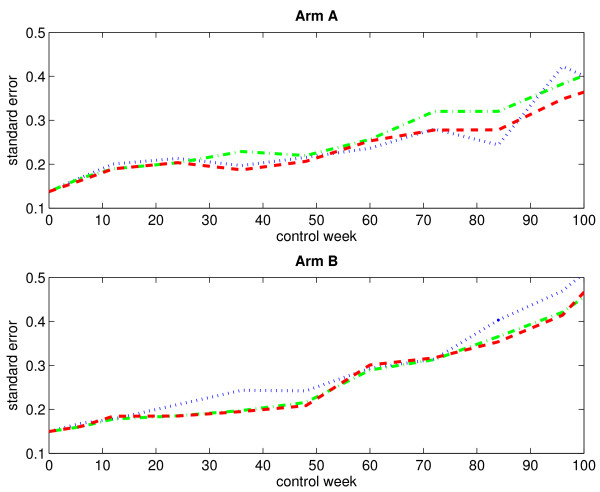
**Empirical standard errors of the estimated mean scores for an immortal cohort (without single imputation)**. The figure displays the empirical standard errors of the estimated mean scores (based on 1000 bootstrap samples) for arm A (upper panel) and arm B (lower panel) when considering an immortal cohort. Single imputation has not been applied. The blue dotted-line curve corresponds to the IPW method, the green dash-dotted-line curve corresponds to the LI method, and the red dashed-line curve corresponds to the MP method.

Figure 3 displays the empirical standard errors of the mean score estimates (based on 1000 bootstrap samples), plotted against time, for both treatment arms when considering an immortal cohort. As expected, the empirical standard errors increase over time. The variability does not seem to differ much between the three estimation methods.

**Figure 4:**
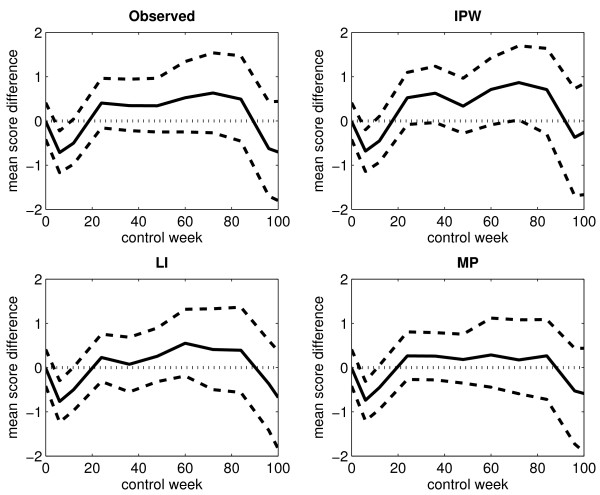
**Differences in the observed and estimated mean scores for an immortal cohort (without single imputation)**. The figure displays the differences in the observed and estimated mean scores between arm A and arm B for the arithmetic average of the observed score values (upper left panel), the IPW method (upper right panel), the LI method (lower left panel) and the MP method (lower right panel) when considering an immortal cohort. Single imputation has not been applied. The black solid-line curve corresponds to the estimated differences, the black dashed-line curves correspond to the upper and lower limits of the 95% percentile interval (based on 1000 bootstrap samples), and the black dotted-line curve corresponds to the zero line.

Figure 4 displays the differences in the mean score estimates between arm A and arm B, plotted against time, when considering an immortal cohort. The corresponding 95% percentile intervals (based on 1000 bootstrap samples) are also shown. The upper and lower percentile limits lying on each side of the zero line corresponds to no significant difference between the two treatment arms. At control week 6, both percentile limits lie on the negative side of the zero line in each of the plots. This indicates a lower mean score in arm A compared to arm B at the end of treatment. In the plot corresponding to the IPW method, the lower percentile limit lies just barely on the positive side of the zero line at control week 72, which indicates a possible higher mean score in arm A. However, this is not supported by the results obtained from the other two estimation methods.

### With single imputation

Table 3 presents the numbers of observed score values, with respect to monotone missingness, for both treatment arms at different control weeks. The corresponding numbers of missing score values are presented in Table 4. By comparing the numbers in Table 1 and Table 3, we see that we get up to 4 and 6 more observed score values at a given control week in arm A and arm B, respectively, when single imputation is applied. Only a few of the score values that are gained have been imputed. The rest of them are available score values that were considered to be missing in the first two analyses where single imputation was not applied, but that now are regarded as observed because of the filling in of non-consecutive missing values preceding them.

**Table 3:** Numbers of observed score values (with single imputation).

		Control week										
		
		0	6	12	24	36	48	60	72	84	96	108
Arm A	Non-imputed	98	73	64	52	48	42	28	27	22	20	15
	
	Imputed	0	0	0	3	1	0	2	0	1	0	0

Arm B	Non-imputed	100	81	73	67	62	47	34	32	24	19	15
	
	Imputed	0	0	0	0	0	3	3	0	0	0	1

The table presents the numbers of observed score values, with respect to monotone missingness, for arm A and arm B at different control weeks. Single imputation has been applied.

**Table 4:** Numbers of missing score values (with single imputation).

		Control week										
		
		0	6	12	24	36	48	60	72	84	96	108
Arm A	Death	0	3	9	23	30	37	48	56	61	66	72
	
	Other causes	0	22	25	20	19	19	20	15	14	12	11

Arm B	Death	0	0	3	13	20	29	42	50	59	62	69
	
	Other causes	0	19	24	20	18	21	21	18	17	19	15

The table presents the numbers of missing score values, with respect to monotone missingness, due to death and other causes for arm A and arm B at different control weeks. Single imputation has been applied.

**Figure 5:**
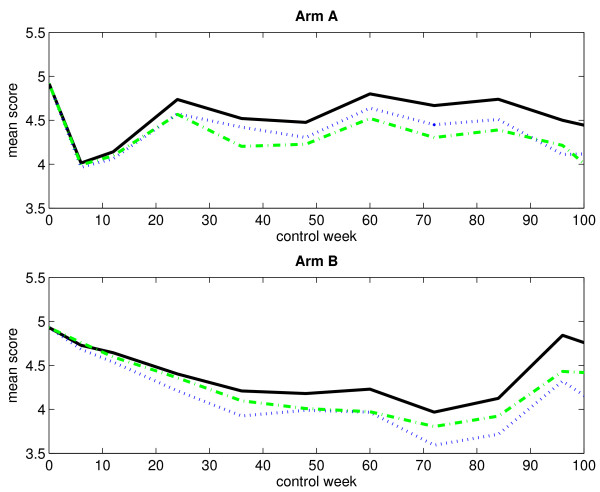
**Observed and estimated mean scores for an immortal cohort (with single imputation)**. The figure displays the observed and estimated mean scores for arm A (upper panel) and arm B (lower panel) when considering an immortal cohort. Single imputation has been applied. The black solid-line curve corresponds to the arithmetic average of the observed score values, the blue dotted-line curve corresponds to the IPW method, and the green dash-dotted-line curve corresponds to the LI method.

**Figure 6:**
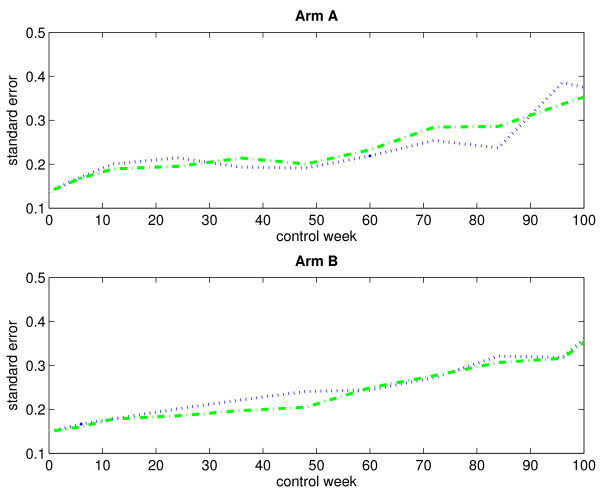
**Empirical standard errors of the estimated mean scores for an immortal cohort (with single imputation)**. The figure displays the empirical standard errors of the estimated mean scores (based on 1000 bootstrap samples) for arm A (upper panel) and arm B (lower panel) when considering an immortal cohort. Single imputation has been applied. The blue dotted-line curve corresponds to the IPW method, and the green dash-dotted-line curve corresponds to the LI method.

Figure 5 displays the mean score estimates, plotted against time, for both treatment arms when considering an immortal cohort. By comparing the curves in Figure 1 and Figure 5, we see that the application of single imputation prior to the data analysis has not changed the observed and estimated mean scores very much. Figure 6 displays the empirical standard errors of the mean score estimates (based on 1000 bootstrap samples), plotted against time, for both treatment arms when considering an immortal cohort. It is evident that single imputation reduces the variability of the estimates.

**Figure 7:**
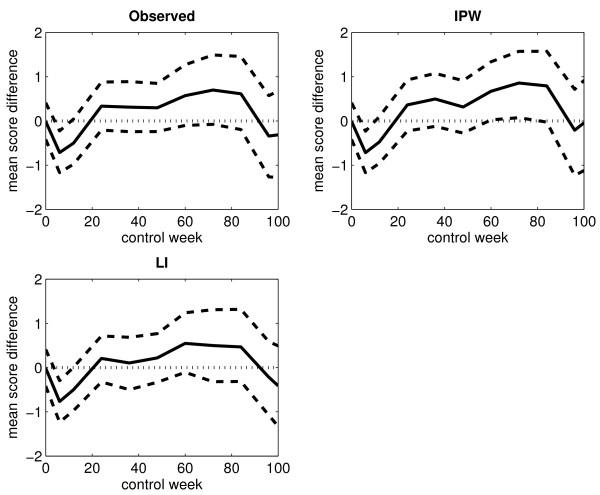
**Differences in the observed and estimated mean scores for an immortal cohort (with single imputation)**. The figure displays the differences in the observed and estimated mean scores between arm A and arm B for the arithmetic average of the observed score values (upper left panel), the IPW method (upper right panel) and the LI method (lower left panel) when considering an immortal cohort. Single imputation has been applied. The black solid-line curve corresponds to the estimated differences, the black dashed-line curves correspond to the upper and lower limits of the 95% percentile interval (based on 1000 bootstrap samples), and the black dotted-line curve corresponds to the zero line.

Figure 7 displays the differences in the mean score estimates between arm A and arm B, plotted against time, when considering an immortal cohort. The corresponding 95% percentile intervals (based on 1000 bootstrap samples) are also shown. The curve patterns resemble the ones displayed in Figure 4.

## Discussion

Results from the data analyses suggest that the true mean score might be overestimated by using the observed mean score, which equals the arithmetic average of the observed score values at a given control week. The most likely reason for this is that the worst patients, that is, the patients with the lowest score values, fail to complete the questionnaire. Thus, higher score values tend to predominate in the data. The initial and sudden drop in the curves of the mean score estimates in the plots corresponding to arm A is in accordance with what might have been expected; the patients in arm A, who received both radiation therapy and chemotherapy, experienced an immediate reduction in mean score, as opposed to the patients in arm B, who received only radiation therapy. However, the difference between the two treatment arms with respect to the mean score seems to diminish over time.

The application of single imputation did not alter the mean score estimates considerably, but the numbers of extra observed score values were indeed quite low. It did, however, lower the empirical standard errors of the mean score estimates. In other words, we gain precision from using single imputation, and this makes our estimates more reliable.

The MP method is certainly the easiest one to implement among the three estimation methods. However, this method, unlike the other two methods, is limited to handle only discrete-valued responses. Further, the IPW method may give more variable estimates and thus less precision [12]. Therefore, we recommend using the LI method in practice when appropriate. This is a good method that is relatively easy to implement. The Matlab^® ^code for the implementation of the methodology considered in this paper is available and can be obtained by contacting the corresponding author.

## Conclusion

Health-related QoL is an important research field of current interest. In medical settings we believe that it is crucial to consider QoL when treatments are being evaluated.

The obtained results from the data analyses corresponding to the three estimation methods agree with one another. Within each treatment arm, the estimated mean scores of self-reported QoL are adjusted downwards compared to the observed mean score. There are significant differences in the estimated mean scores of self-reported QoL between arm A and arm B at the end of treatment.

## Competing interests

The authors declare that they have no competing interests.

## Authors' contributions

NG and OOA conceived the methodological approach. NG performed the statistical analysis and drafted the manuscript. TGS contributed to the methodology. SA, PFB and SS were responsible for the clinical trial. ABJ performed data quality assurance. All authors read and approved the final manuscript.

## Pre-publication history

The pre-publication history for this paper can be accessed here:

http://www.biomedcentral.com/1471-2288/9/28/prepub
